# Longitudinal study of influenza A virus circulation in a nursery swine barn

**DOI:** 10.1186/s13567-017-0466-x

**Published:** 2017-10-10

**Authors:** Juliana B. Ferreira, Helena Grgić, Robert Friendship, Greg Wideman, Éva Nagy, Zvonimir Poljak

**Affiliations:** 1 Department of Population Medicine, University of Guelph, Guelph, ON Canada; 2 South-West Veterinary Services, Stratford, ON Canada; 3 Department of Pathobiology, University of Guelph, Guelph, ON Canada

## Abstract

Commercial production of swine often involves raising animals in large groups through the use of multi-stage production systems. In such systems, pigs can experience different degrees of contact with animals of the same or different ages. Population size and degree of contact can greatly influence transmission of endemic pathogens, including influenza A virus (IAV). IAV can display high genetic variability, which can further complicate population-level patterns. Yet, the IAV transmission in large multi-site swine production systems has not been well studied. The objectives of this study were to describe the IAV circulation in a multi-source nursery facility and identify factors associated with infection in nursery pigs. Pigs from five sow herds were mixed in one all-in/all-out nursery barn, with 81 and 75 pigs included in two longitudinal studies. Virus isolation was performed in Madin-Darby canine kidney cells and serology was performed using hemagglutination inhibition assays. Risk factor analysis for virological positivity was conducted using logistic regression and stratified Cox’s regression for recurrent events. In Study 1, at ≈30 days post-weaning, 100% of pigs were positive, with 43.2% of pigs being positive recurrently over the entire study period. In study 2, 48% of pigs were positive at the peak of the outbreak, and 10.7% were positive recurrently over the entire study period. The results suggest that IAV can circulate during the nursery phase in an endemic pattern and that the likelihood of recurrent infections was associated in a non-linear way with the level of heterologous (within-subtype) maternal immunity (*p* < 0.05). High within-pen intracluster correlation coefficients (> 0.75) were also observed for the majority of sampling times suggesting that pen-level factors played a role in infection dynamics in this study.

**Electronic supplementary material:**

The online version of this article (doi:10.1186/s13567-017-0466-x) contains supplementary material, which is available to authorized users.

## Introduction

Influenza A virus (IAV), an enveloped negative-stranded RNA virus, belongs to the family *Orthomyxoviridae* and is subtyped based on two surface glycoproteins: hemagglutinin (HA) and neuraminidase (NA) [1]. Influenza A viruses are the infectious agents frequently involved in acute respiratory disease outbreaks in pigs [2], with three subtypes (H1N1, H3N2, and H1N2) endemic worldwide [3]. In addition, IAV is one of the agents commonly found in porcine respiratory disease complex along with other viral and bacterial pathogens [4]. Respiratory diseases in pigs can occasionally result in severe outcomes such as mortality and is commonly linked with reduction in the efficiency of feed conversion, growth retardation, and reduction in carcass quality [5].

As in many other species, infection in individual pigs is considered to be relatively simple with short duration of infectiousness and quick development of active immunity [6, 7]. Clinical signs and pathological lesions can exist for some time after the infection has been resolved [8]. At the population level, outbreaks of influenza in pigs are usually recognized by high morbidity and low mortality with sudden appearance of respiratory signs and also by quick recovery of sick animals [7]. Transmissibility of influenza A virus in pigs can be influenced by factors such as age, immunity, vaccination status, and presence of maternal antibodies among other factors [9, 10].

Since the emergence of the 2009 pandemic H1N1 virus in swine populations [11], a number of novel reassortant variants of IAV has been reported in pigs across the world [1, 12–16], including Canada [17–21]. Several studies have additionally reported exposure of pigs to more than a single strain or subtype, either cumulatively or concurrently [22–24]. Such existence of multiple strains introduces new uncertainties in our understanding of how influenza A viruses circulate in swine populations, and complicates control measures including the design of vaccination protocols in individual herds or entire multi-site production systems. This issue could be further amplified in production systems where animals from different sources are mixed as a part of regular operating procedures. Thus, the objectives of this study were to describe patterns of IAV infection in pigs after weaning in multi-source nursery herds and determine factors that contribute to infection with IAV. The objectives were fulfilled by conducting two single-cohort studies at the pig level.

## Materials and methods

### General overview

The pig farm selected for this study was a nursery only site located in southern Ontario, with a total capacity of 4000 animals housed in two separate barns with equal capacity. The farm was a part of a multi-site swine operation with directed flow that included five sow herds. Pigs from all five sow sources were weaned at approximately 19 days of age, and transported into one nursery barn in a given week where they formed a nursery batch. Thus, the two barns on this site were filled and emptied over a total of 2 weeks, meaning that each barn was operating at full capacity 1 week after being emptied, and the farm and site facility were operated on an all-in/all-out (AIAO) basis. The production system had a history of ongoing respiratory disease that was attributed to infection with IAV, and this included lower than expected average daily gain during the nursery phase. The site was included in this study because of: (1) convenient access, (2) site outline that allowed practicing appropriate biosecurity measures in an efficient manner, and (3) respiratory clinical signs that were attributed to IAV in multiple pig batches before the study started. In the study barn, two pig-level longitudinal studies were performed in two distinct periods: Study 1 between November 18^th^ 2013 and January 9^th^ 2014 and Study 2 between April 4^th^ 2014 and May 29^th^, 2014, respectively. Sows from the sow herds were vaccinated with a commercial multivalent vaccine (Flu Sure^®^XP, Zoetis, Canada) in Study 1 and with an autogenous vaccine based on H3N2 strains in Study 2.

### Study population

The study barn, selected for performing the 2 trials, had four equally-sized rooms, each with a separate air flow, and each holding approximately 500 pigs in 24 pens, for a total capacity of approximately 2000 pigs. During both studies, the barn accommodated a total of 238 and 588 piglets from sow-herd 1, 805 and 769 from sow-herd 2, 327 and 359 from sow-herd 3, 245 and 130 from sow-herd 4, and 290 and 91 from sow-herd 5 for Studies 1 and 2, respectively. Pigs were mixed upon arrival in these rooms with pigs from three to four sources in each of the rooms.

For the initial virological test, 400 piglets for Study 1 (80 per sow source) and 300 piglets for Study 2 (60 per sow source) were selected for nasal swabbing using a convenience sampling. Nasal samples were obtained using sterile polyester swabs (Pur-Wraps^®^, Puritan, Guilford, ME, USA) within the first 2 h of arrival at the nursery. In each study, at least 15 pigs per sow source for a total of 81 and 75 in Studies 1 and 2, respectively, were included for longitudinal study. These pigs were ear tagged and blood sampled at the beginning, and sampled using nasal swabs on a weekly basis until the end of the studies. The swabs were placed in 2 mL of phosphate-buffered saline (PBS), and kept on ice for transport and then frozen at −80 °C until tested. Blood samples were collected in 10 mL serum tubes (BD Vacutainer^®^, Franklin Lakes, NJ, USA), centrifuged at 390 rpm for 15 min at 5 °C (Centra CL3R^®^, Thermo Electron Corporation, USA) and sera were extracted and kept at −20 °C until serological testing. Sample size of 80 and 60 animals per sow-source was sufficient to detect infection at entry to nursery of approximately 3.5 and 5%, respectively with 95% confidence under assumption of 95% test sensitivity and perfect specificity in a population with a maximum of 500 animals. Overall, sample size of 400 and 300 animals at the beginning was sufficient to detect prevalence of 0.5 and 1%, respectively. Sample size of 75 animals was sufficient to detect circulation of influenza virus at 4% using identical assumptions. The study was approved by the Animal Care Committee of the University of Guelph.

### Detection and identification of influenza A virus

Presence of IAV from nasal swabs was assessed by isolation and propagation of the virus in Madin-Darby canine kidney (MDCK) cells with added trypsin according to standard protocol [25]. The choice of this technique was based on recent studies that showed that MDCK cell lines proved to be highly sensitive for IAV isolation [26, 27]. Virus replication was confirmed based on the cytopathic effect (CPE) produced in the cells and also assessed by hemagglutination assay according to standard protocol [25]. Selected isolates from both studies were submitted for sequencing as reported elsewhere [28]. Illumina Sequencing By Synthesis (SBS) was conducted by the Clinical Genomics Centre, the UHN/MSH Gene Profiling Facility (Mount Sinai Hospital, Toronto, ON, Canada). Sequencing was performed with an Illumina Miseq (Illumina, San Diego, CA, USA) for 300 cycles of paired-end sequencing run. The data pipeline was performed using the Illumina sequence analysis software, Casava (Version 1.8.2). For the purposes of this study, samples from pigs with repeated positive samples were sequenced, and the nucleotide sequences of the complete hemagglutinin gene were analyzed and compared to current strains that circulate in North America and Ontario. Five isolates from Study 1, and four isolates from Study 2 were utilized. The consensus nucleotide sequences were aligned using Clustal W algorithm and the distance matrix was calculated using Juke-Cantor method; following which the neighbor-joining method was used to construct the dendrogram, with 1000 bootstrap iterations to evaluate the tree reliability. Geneious 9 was used to conduct phylogenetic analysis [29].

### Serology

In order to determine the level of maternally-derived antibodies (MDA) at entry to the nursery, sera were analyzed by hemagglutination inhibition (HI) assay according to standard protocol [30] with four hemagglutinin units per well. Sera were heat inactivated for 30 min at 56 °C and treated with a 20% kaolin solution to remove non-specific inhibitors of hemagglutination. Cut-off of HI was set to ≥ 1:40 as previously reported [30]. Titers were then divided by 10 and log_2−_ transformed for the purposes of statistical analysis. Six previously isolated swine influenza strains [17, 18] were used for HI: A/SW/ON/103-18/11/H3N2, A/SW/ON/104-25/12/H3N2, A/SW/ON/115-2/12/H3N2, A/SW/ON/68/12/H1N2, A/SW/ON/84/12/H1N1, A/SW/2/81/H1N1 and throughout the article we will refer to them as H3N2_A, H3N2_B, H3N2_C, H1N2, H1N1_P, and H1N1_C, respectively. The selection of H3N2 strains (A, B, C) was based on the isolation and identification of those in Ontario farms [18, 28], and the selection of H1N1 (C, P) and H1N2 was based on the fact that H1N1_P was the prevalent strain circulating in swine farms in Ontario and also due to the isolation and identification of H1N1_C and H1N2 based on the recent study conducted in Ontario herds [17]. Also, the two viruses identified in these longitudinal studies were used for HI: A/SW/ON/72-7-8/2014/H3N2 (Study 1) and A/SW/ON/148-9/2014/H1N1 (Study 2) and throughout the article we will refer to them as H3N2_home and H1N1_home, respectively.

Sera were also analyzed by enzyme-linked immunosorbent assay (ELISA) according to the manufacturer’s instruction for *Mycoplasma hyopneumoniae* and porcine reproductive and respiratory syndrome virus (PRRSV) (IDEXX *M. hyo* and IDEXX PRRS X3, 2011).

### Statistical analysis

In both studies, levels of MDA at entry to the nursery and serological positivity for *M. hyponeumoniae* and PRRSV were used as primary exposures of interest. In addition, the level of MDA at initial sampling against different H3 influenza viruses, and different H1 influenza viruses was analyzed separately by hierarchical agglomerative cluster analysis performed in R 3.1.0 (The R Foundation for Statistical Computing) using FactoMineR package [31]. Grouping of pigs into clusters with similar level of MDA was based on different inputs including: (1) HI titers of H3N2 viruses that were heterologous to the resident H3N2 virus, which includes H3N2_A, H3N2_B, H3N2_C viruses and (2) HI titers of all H3N2 viruses that includes H3N2_A, H3N2_B, H3N2_C, and H3N2_home viruses. The resulting groups from these two cluster analyses were used as separate categorical risk factors. Also, information on gender, sow source, and room were recorded and analyzed as risk factors.

Descriptive statistics were generated for each variable and correlation was tested using the Spearman correlation coefficient.

#### Random effect logistic regression models

Mixed effect logistic regression model with pen as a random effect on intercept was used to evaluate development of IAV shedding over time, and its association with exposures of interest. Models were set separately for the two longitudinal studies. Models were built in a forward stepwise fashion where the linear, quadratic and cubic effect of time on the logit of IAV positivity were evaluated. This was followed by inclusion of linear and quadratic form of the log2 HI titers, or appropriate binary variables. Finally, interaction between the effect of time and exposures of interest was evaluated for statistical significance. In addition, at each sampling week when data allowed, an empty model was constructed using random effect logistic regression model with pen as a random effect on intercept. The intra-cluster correlation coefficient (ICC) was determined using a method described elsewhere [32].

#### Logistic regression

In addition, results of repeated virological testing per each pig were aggregated to a pig-level and animals were then categorized into distinct categories: (1) pigs that were never positive during the study period; (2) pigs that were positive only once during the study period; (3) pigs that were subsequently virologically positive on two or more repeated samplings; (4) pigs that were recurrently positive on more than one occasion, but with one or more virologically negative samplings between the positive samplings. Then, pigs in class 4 were considered as positive for recurrent infection, and pigs in the other three groups were considered as negative for the recurrent infection. Ordinary logistic regression was performed for the same set of potential risk factors as in the random effect logistic regression model. Models were evaluated as described elsewhere [33].

#### Stratified Cox’s regression for recurrent events

Risk factors for the recurrent virological positivity over time were also analyzed using the Prentice, Williams and Peterson conditional probability (PWP-CP) approach [34]. Briefly, the approach is based on the Cox’s regression that accounts for recurrent events. The PWP-CP model analyzes the ordered multiple events by stratification which is based on the prior number of episodes during the follow-up period. A stratum variable is used to keep track of the event number. Interaction between strata and titer for each virus analyzed was evaluated in order to evaluate whether association between exposures of interest was different for different events. The PWP-CP is also a conditional model in which all participants are at risk for the first stratum, but only those with an event in the previous stratum are at risk for the successive one. As such, every time a pig had an event and recovered from it, this same pig was at risk for a second event, i.e. an influenza episode (event) had to be preceded and followed by a time period without influenza to be considered as conditional. Models were compared during the model-building process using likelihood ratio test and Akaike’s information criterion. The overall model fit was investigated based on the graphical analysis of residuals.

Descriptive analysis was also performed for other environmental and population-level measurements obtained during the study period, such as: mortality rate and microclimate inside the barn including: relative humidity (RH), temperature and absolute humidity (AH). Microclimate measurements were recorded every five minutes (HOBO^®^ data logger, Onset Computer Corporation, Bourne, MA, USA) in each room for a period of 7 weeks, which comprises the period that pigs stayed in each of the three rooms in the barn. Original data were exported as a comma separated value (CSV) file and imported to a statistical program for further processing and analysis. AH was calculated based on previous references [35, 36]. Statistical analyses were conducted at the pig level using STATA IC 13 (StataCorp LP, College Station, TX, USA). Microclimate conditions, originally measured every 5 min over the entire study period, were summarized through a single measure of descriptive statistics such as mean, standard deviation (SD), minimum, and maximum.

## Results

**Figure 1:**
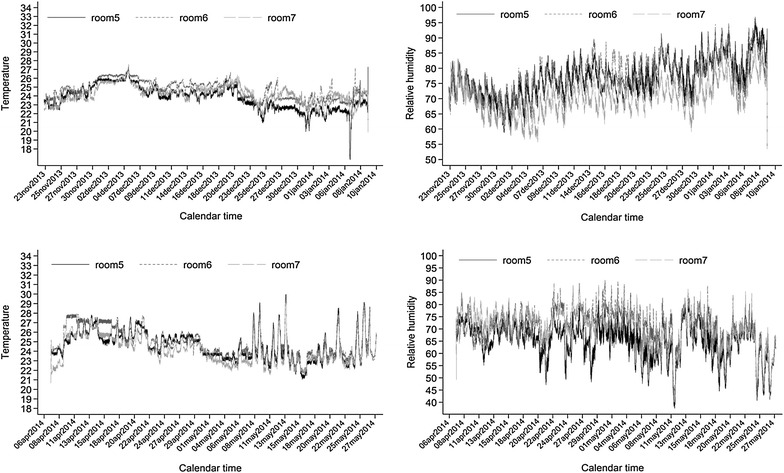
**Daily changes in relative humidity and temperature in each room during study periods.**

Overall mortality in the barn was 1.8 and 1.9% for Studies 1 and 2, respectively. Two pigs died in each longitudinal study. Pigs in Study 1 died on December 27^th^, 2013 (week 6) and January 7^th^ (week 8), 2014, with peracute illness resulting in sudden death. In Study 2, study animals died on May 16^th^ (week 6), with peracute illness resulting in sudden death, and on May 20^th^ 2014 (week 7), with clinical signs consistent with infection with *Streptococcus suis*. As such, results obtained from these animals were included in the pig-level analysis, but their measurements were considered as missing for repeated measures and censored at appropriate times for the purposes of survival analysis. Descriptive analysis of all variables, from both studies, is presented in Table 1. For the temperature and humidity, mean, minimum, and maximum for temperature, RH and AH were also evaluated (Table 1 and Figure 1). Measurements for temperature and humidity were collected every 5 min during study periods.

**Table 1:** **Descriptive statistics of the main quantitative measurements used as explanatory variables in regression models**

Variable	Study 1^a^	Study 2^b^
	Median (IQR)	Median (IQR)
H3N2_A^c^	10 (15)	40 (40)
H3N2_B^c^	40 (60)	160 (80)
H3N2_C^c^	20 (30)	80 (120)
H3N2_home^d^	40 (140)	80 (120)
H1N2^e^	40 (20)	80 (120)
H1N1_C^f^	10 (15)	10 (15)
H1N1_P^g^	10 (15)	20 (30)
H1N1_home^d^	40 (70)	20 (30)

Variable	Study 1^a^	Study 2^b^
	Mean (SD)	Mean (SD)
Room5		
RH (%)	76.8 (6.3)	65.8 (7.4)
Temp (°C)	23.7 (1.3)	24.5 (1.5)
AH (mb)^h^	23.1 (1.8)	20.8 (2.7)
Room6		
RH (%)	75.4 (10.0)	71.9 (7.3)
Temp (°C)	24.3 (1.7)	24.8 (1.5)
AH (mb)^h^	23.9 (4.0)	23.2 (2.7)
Room7		
RH (%)	68.6 (9.2)	69.0 (10.0)
Temp (°C)	23.8 (1.5)	23.7 (2.2)
AH (mb)^h^	21.0 (3.5)	21.1 (4.3)

Median and interquartile range, or mean and standard deviation were used, as it was deemed appropriate.

^a,b^Two distinct longitudinal studies of IAV circulation were performed in nursery pigs.

^c^Different H3N2 variants broadly classified into cluster 4 of H3N2 swine influenza A virus and isolated in Ontario in 2012 and used in the hemagglutination inhibition assay.

^d^H3N2 and H1N1 viruses detected in the study and used as antigens in the hemagglutination inhibition assay.

^e^H1N2 with hemagglutinin of the 2009 pandemic lineage and neuraminidase of the Cluster 4 H3N2 IAV-S.

^f^H1N1 IAV-S broadly classified as the classical swine H1N1 virus used in hemagglutination inhibition assay.

^g^H1N1 IAV-S of the 2009 H1N1 pandemic lineage used in hemagglutination inhibition assay.

^h^Absolute humidity expressed as milibar (MB).

### Viral shedding

#### Study 1

**Figure 2:**
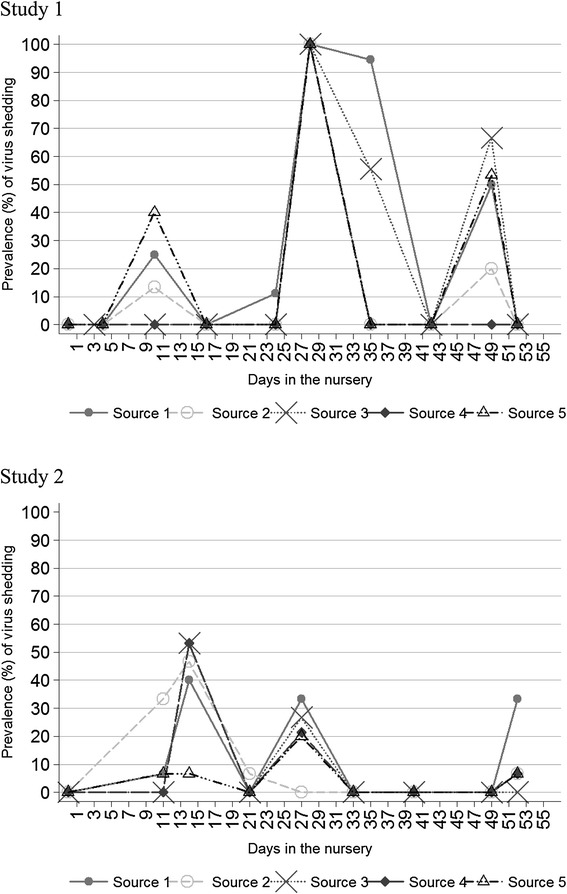
**Overall positivity of IAV by sow source based on isolation and confirmation of the virus.** Isolated viruses in Study 1 and Study 2 were H3N2 and H1N1, respectively.

**Figure 3:**
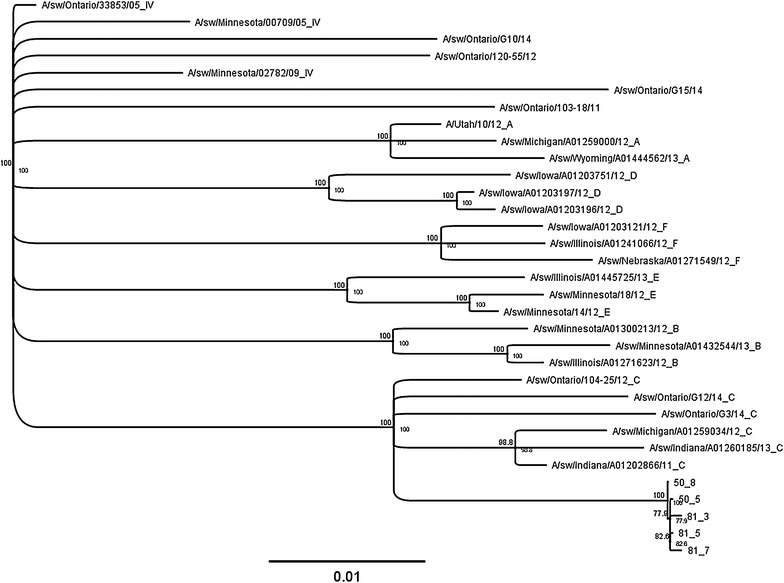
**Phylogenetic tree based on nucleotide sequence of the entire** **hemagglutinin gene of five H3N2 viruses detected in Study 1** **and relevant standards representing original cluster IV H3N2 viruses, six subgroups within cluster IV** (**A**–**F**)**, and recent uncategorized H3N2 Ontario viruses.** The tree is built using Neighbor-Joining method and its reliability is estimated using 1000 bootstraps. Note that standard names have been shortened and modified so that the end of the name after underscore represent the cluster number (IV), or subgroup designation (A–F). The study viruses are labeled with the pig identification number, followed by sampling number. The scale represents number of substitutions per site.

Three distinct modes of viral shedding were observed in the nursery barn over a period of 53 days with the peak prevalence of 100% detected at 29 days of the study (Figure 2, Study 1). Pigs sourced from more than one sow-herd were detected to shed the virus on more than 1 weekly sampling, with variability in the extent of number of positive pigs observed among sources (Table 2, Study 1), and with some of the pigs being positive four times. Overall, based on different analysis (data not shown), it was observed that out of 81 pigs, 38 (46.9%), 8 (9.9%), and 35 (43.2%) were classified in category 2, 3, and 4 as shown in the logistic regression methods, respectively. The time period observed between the first and last shedding, i.e. recurrent infections, in individual pigs was between a minimum of 7 and maximum of 39 days. A subset of isolated viruses was sequenced and all isolates were characterized as identical viruses herein named A/SW/ON/72-7-8/2014/H3N2 (unpublished data). From the sequence analysis it is clear that viruses identified in this study formed a homogeneous group of viruses when compared to the used standards (Figure 3). The minimum similarity among the studied viruses was 99.9% and maximum was 100%. Furthermore, viruses from the same pigs that were repeatedly positive showed high degree of homology (i.e. 100%) despite extended lags between samplings in some cases (Figure 3). The viruses isolated in this study were categorized into subgroup C of cluster IV H3N2 virus, which was documented to be the most common Ontario strain in a recent study [28]. However, this strain is distinct from strains reported in a previous study and at the level of nucleotide sequence showed similarity of 93.8% with the closest subgroup C isolate which was used as a standard in this study.

**Table 2:** **Frequency of IAV shedding in nursery pigs in two longitudinal studies stratified by sow source**

N detections^a^	Source 1	Source 2	Source 3	Source 4	Source 5	Total N	Total %	(95% CI)
Study 1								
0	0	0	0	0	0	0	0.0	
1	0	12	4	15	7	38	46.9	(35–58)
2	7	1	6	0	2	16	19.7	(11–30)
3	9	2	8	0	6	25	30.8	(21–42)
4	2	0	0	0	0	2	2.4	(1–8)
Total^b^	18	15	18	15	15	81^c^	100.0^c^	

N detections^a^	Source 1	Source 2	Source 3	Source 4	Source 5	Total N	Total %	(95% CI)
Study 2								
0	4	6	4	4	9	27	36.0	(25–47)
1	6	4	10	10	6	36	48.0	(36–59)
2	4	5	1	1	0	11	14.6	(7–24)
3	1	0	0	0	0	1	1.3	(1–7)
4	0	0	0	0	0	0	0.0	
Total^b^	15	15	15	15	15	48^c^	64.0^c^	(52.1–74.8)^c^

^a^Number of times that virus was isolated from individual nursery pigs.

^b^Total number of pigs included in the study by sow source.

^c^Represents number of pigs that were positive at least once in the study. May not be a sum of this column or row.

#### Study 2

**Figure 4:**
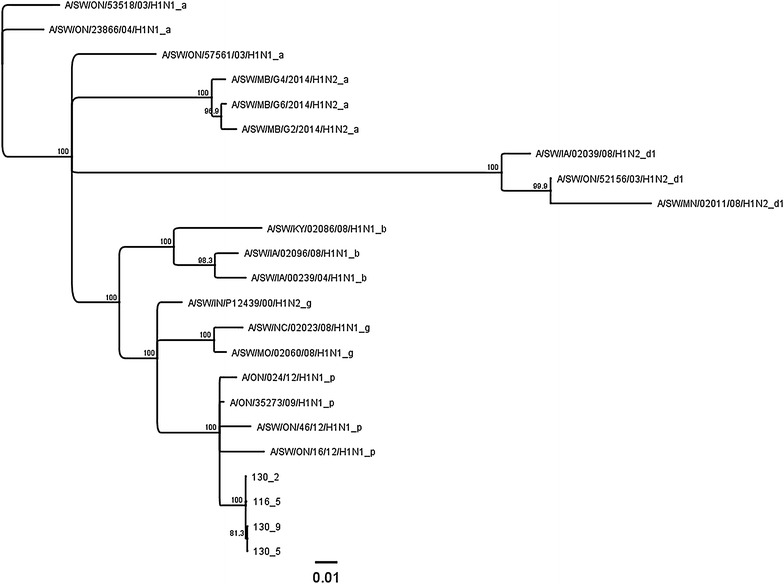
**Phylogenetic tree based on nucleotide sequence of the entire** **hemagglutinin gene of four H1N1 viruses detected in Study 2 and relevant standards representing different clusters of H1 viruses [a** **=** **alpha, b** **=** **beta, d1** **=** **delta1, g** **=** **gamma, and p** **=** **pandemic (pdm(H1N1)].** The tree is built using Neighbor-Joining method and its reliability is estimated using 1000 bootstraps. Note that standard names have been shortened and modified so that the end of the name after underscore represent the cluster designation (a–p). The study viruses are labeled with the pig identification number, followed by sampling number. The scale represents number of substitutions per site.

Three distinct modes of viral shedding were observed in the nursery barn over a period of 53 days with the peak prevalence of 53% detected at 14 days of the study (Figure 2, Study 2). Pigs sourced from more than one sow-herd were detected to shed the virus on more than 1 weekly sampling, with variability in the extent of number of positive pigs observed among sources (Table 2, Study 2). Overall, based on different analysis (data not shown), it was observed that out of 75 pigs, 27 (36%), 36 (48%), 4 (5.3%), and 8 (10.6%) were classified in category 1, 2, 3, and 4 as shown in the logistic regression methods, respectively. The time period observed between the first and last shedding, i.e. recurrent infections, in individual pigs was between a minimum of 3 and maximum of 41 days. A subset of isolated viruses was sequenced and all isolates were characterized as identical viruses herein named A/SW/ON/148-9/2014/H1N1 (unpublished data). From the sequence analysis it is clear that the H1N1 viruses identified in this study also formed a homogeneous group when compared to the standards used (Figure 4). The minimum similarity among the study viruses was 99.8% and maximum was 100%. Similarly to Study 1, viruses detected in the same pig that was repeatedly positive showed high degree of homology (i.e. 100%) again despite lags between samplings (Figure 4). The viruses isolated in this study were categorized as a pdm(H1N1), which was documented as a frequent virus circulating in Ontario swine populations based on recent studies [17].

All of the pigs (400 and 300 in Study 1 and 2, respectively) sampled during the first 2 h after arrival at the nursery for both studies were negative for IAV.

### Serology

#### Study 1

**Figure 5:**
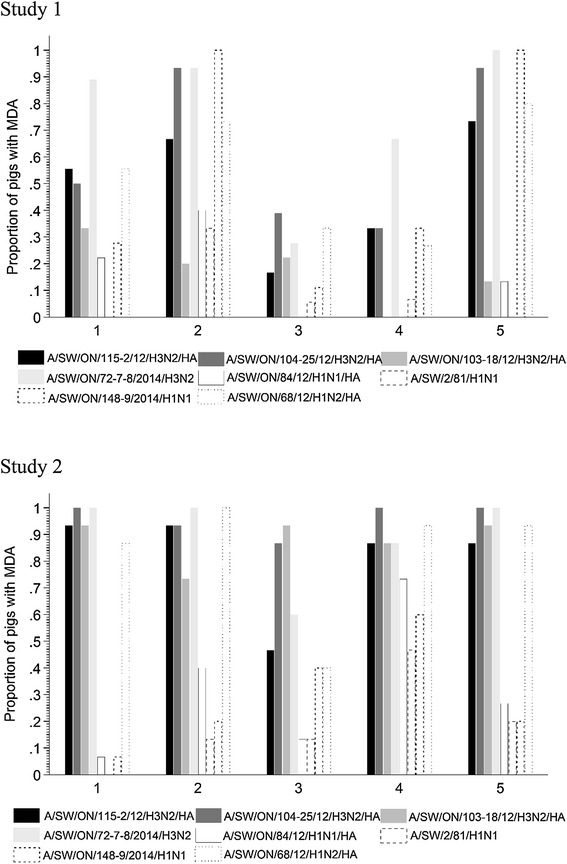
**Proportion of pigs positive for eight IAVs at weaning, stratified by sow source** (**1**–**5).** Antibodies against influenza A viruses were analyzed by hemagglutination inhibition assay where the cut-point for positivity was assumed to be titers over 1:40.

Sera collected upon arrival to the nursery suggested variability in the level of MDA for all 8 viruses tested (Figure 5, Study 1 and Table 1) in all five sources (1–5). In general, piglets from sources 3 and 4 had the lowest proportion of pigs positive for MDA against several IAV strains. Results showed a positive correlation amongst all titers with 9 Spearman correlation coefficients ranging between 0.5 and 0.8. Titers for two viruses, H3N2_C and H3N2_A, were both highly correlated with H1N2 (>0.8). An additional table file shows this in more detail (see Additional file 1). Two clusters were created on the basis of the agglomerative hierarchical cluster analysis of sera for heterologous and all H3N2 viruses (Table 3), and three clusters were created for viruses with different titers for MDA for viruses containing H1 hemagglutinin. The cluster relevant for further analysis was the heterologous H3N2 cluster, in which the groupings were designated as “high heterologous” (19.7%) and “low heterologous” (80.2%). The high heterologous cluster had median HA titers for H3N2_A, H3N2_B, and H3N2_C of 1:40 (IQR = 40), 1:80 (IQR = 100), and 1:80 (IQR = 60), respectively, whereas low heterologous had median HA titers for H3N2_A, H3N2_B, and H3N2_C of 1:10 (IQR = 15), 1:40 (IQR = 20), and 1:20 (IQR = 35), respectively. Detailed statistics for other clusters are not shown as they were not associated with the final outcome (data not shown). ELISA results showed that 67 pigs (82.7%) had titers for *M. hyopneumoniae* and 25 (30.9%) for PRRSV.

**Table 3:** **Number of pigs (%) with detectable antibodies at weaning and description of other categorical variables**

Variable	Study 1^a^	Study 2^b^
	N (%)	N (%)
H3N2_A + ve^c^	15 (18.5)	66 (88.0)
H3N2_B +ve^c^	49 (60.5)	72 (96.0)
H3N2_C +ve^c^	39 (48.1)	61 (81.3)
H3N2_home +ve^d^	60 (74.1)	67 (89.3)
H1N2 +ve^e^	43 (53.1)	62 (82.8)
H1N1_C +ve^f^	7 (8.6)	14 (18.7)
H1N1_P +ve^g^	15 (18.5)	24 (32.0)
H1N1_home +ve^d^	42 (51.8)	22 (29.3)
Cluster_H3N2_Heterologous^h^		
Cluster 1 (low heterologous)	65 (80.2)	45 (60.0)
Cluster 2 (high heterologous)	16 (19.7)	24 (32.0)
Cluster 3 (high heterologous)	–	6 (8.0)
PRRSV +ve^i^	25 (30.9)	14 (18.7)
Mhyo +ve^j^	67 (82.7)	64 (85.3)
Female	52 (65.8)	37 (49.4)
Male	27 (34.2)	38 (50.7)
Room5	45 (55.6)	30 (40.0)
Room6	9 (11.1)	30 (40.0)
Room7	27 (33.3)	15 (20.0)

^a,b^Two distinct longitudinal studies were performed in nursery pigs.

^c^Different H3N2 variants broadly classified into cluster 4 of H3N2 swine influenza A virus and isolated in Ontario in 2012 and used in the hemagglutination inhibition assay.

^d^H3N2 and H1N1 viruses detected in the study and used as antigens in the hemagglutination inhibition assay.

^e^H1N2 with hemagglutinin of the 2009 pandemic lineage and neuraminidase of the Cluster 4 H3N2 IAV-S.

^f^H1N1 influenza A virus (IAV-S) broadly classified as the classical swine H1N1 virus used in hemagglutination inhibition assay.

^g^H1N1 influenza A virus (IAV-S) of the 2009 H1N1 pandemic lineage used in hemagglutination inhibition assay.

^h^Groupings based on cluster analysis of maternally derived antibodies for heterologous strains of H3N2 viruses (labeled here with^c^).

^i^Positive for maternally derived antibodies for PRRSV.

^j^Positive for maternally derived antibodies for *Mycoplasma hyopneumoniae.*

#### Study 2

Sera collected on the first day in the nursery indicated that high proportion of pigs among all five sources (1–5) had MDA for different H3N2 strains used as antigens in the HI assay (Figure 5, Study 2 and Table 1). In contrast, MDA against H1N1 strains were generally lower. Interestingly, a considerable proportion of pigs among all sources were positive for MDA against the H1N2 strain. Results showed positive and negative correlation amongst all titers with negative Spearman correlation coefficients ranging between 0.1 and −0.08. Positive results showed 12 Spearman correlation coefficients ranging between 0.5 and 0.8. The titers for antibodies against one virus, H1N2, were highly correlated with titers against H3N2_C (> 0.8). An additional table file shows this in more detail (see Additional file 1). Three clusters were created on the basis of the agglomerative hierarchical cluster analysis of sera for heterologous and all H3N2 viruses (Table 3), and also for viruses with different titers for MDA for viruses containing H1 hemagglutinin. Detailed statistics for clusters are not shown as they were not associated with the final outcome (data not shown). Results of ELISA showed that 64 pigs (85.3%) had titers for *M. hyopneumoniae* and 14 (18.7%) for PRRSV.

#### Random effect logistic regression

**Figure 6:**
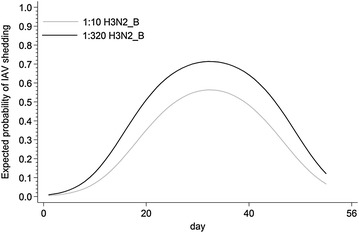
**Predicted probability of having IAV infection during the nursery period.** Prediction was calculated for a pig with low (1:10) and a pig with high (1:320) level of antibodies for H3N2_B virus (A/SW/ON/104-25/12/H3N2) at weaning based on the random effect logistic regression.

Results for the mixed effect logistic regression for Study 1 are presented in Table 4. Out of all variables representing the antibody status at weaning that were evaluated in this analysis, only the log2 titers for H3N2_B virus were associated with the probability of IAV shedding in individual pigs over time (Table 4). However, no interaction between titers and time was detected, suggesting that the effect of MDA at weaning was consistent over the study period. The expected probability of viral positivity for pigs with different levels of MDA at entry to the nursery is presented in Figure 6. Based on the model, pigs with higher MDA titers for H3N2_B are more likely of shedding the virus throughout the study period (Figure 6). Source herd was also associated with the probability of IAV shedding in individual pigs over time (Table 4). No explanatory variables were associated with IAV positivity using this approach in Study 2 (*p* > 0.05).

**Table 4:** **Two logistic regression models for shedding of IAV in individual pigs over time in Study 1**

Variable	Coefficient	(95% CI)	*p* value
Model 1			
Time (days)	0.37	(0.28, 0.45)	<0.01
Time (days)^2 a^	−0.01	(−0.006, −0.004)	<0.01
H3N2_B^b^	−0.31	(−0.59, −0.02)	0.03
H3N2_B^2 a,b^	0.08	(0.01, 0.14)	0.01
Model 2			
Time (days)	0.37	(0.28, 0.45)	<0.01
Time (days)^2 a^	−0.01	(−0.006, −0.004)	<0.01
Source sow herd^c^			<0.01^**\\**^
Source 1	1.46	(0.78, 2.13)	0.01
Source 2	0.36	(-0.38, 1.1)	0.34
Source 3	1.09	(0.41, 1.77)	0.01
Source 4	Baseline	–	–
Source 5	0.87	(0.16, 1.59)	0.01

Model 1 depicts association of viral shedding with maternally derived antibodies for (f A/SW/ON/104-25/12/H3N2 (H3N2_B). Logistic regression with pig as a random effect on intercept was used. The Model 2 details association with the source herd.

^**\\**^p-value obtained by testing categorical variable using a partial likelihood test.

^a^Quadratic effect of the variable.

^b^The original titer divided by 10 and then log2 transformed.

^c^Coefficients are adjusted for the linear and quadratic effect of time.

Results of the empty models showed that for those weeks where the ICC was calculated, high proportion in the variation of the outcome at each sampling time resided at the pen level. Weeks 3, 5, 7, and 9 in Study 1 showed an ICC of 0.76, 0.99, 0.96, and 0.86, respectively. Weeks 2, 3, 5, and 9 in Study 2 showed an ICC of 0.88, 0.55, 0.85, and 0.75, respectively.

#### Logistic regression

**Figure 7:**
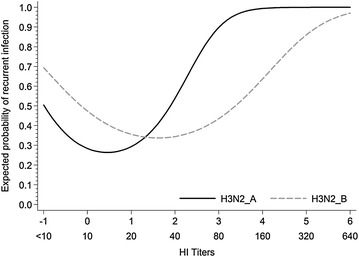
**Predicted probability of having recurrent IAV H3N2 infection during the nursery period.** Prediction was calculated for pigs with different HI titers at weaning for H3N2_A (A/SW/ON/103-18/11/H3N2) and H3N2_B (A/SW/ON/104-25/12/H3N2) viruses based on logistic regression model.

Results for the logistic regression model are presented on Table 5 for both studies. Results showed that the presence of initially high titers for MDA for H3N2_A and H3N2_B were associated with the likelihood of recurrent infection in a non-linear fashion (*p* < 0.05). Figure 7 depicts that pigs with MDA at weaning above the cutpoint (i.e. >40) had higher probability of recurrent infections than pigs that were at or below that cutpoint (i.e. 10–40). Nonetheless, pigs detected with titers below the detection limit (i.e. <10) are expected to have slightly higher likelihood of recurrent infection compared to the pigs with titers ranging from 10 to 40. In addition, presence of MDA for H1N1_home appeared to play a role in the odds of having recurrent infection when evaluated independently.

**Table 5:** **Univariable associations for recurrent IAV shedding using logistic regression and Cox’s regression for recurrent events**

Variable	Ordinary logistic regression			PWP-CP		
	OR	95% CI	*p*-value	HR	95% CI	*p*-value
Study 1^a^						
H3N2_A^c,d^	0.63	0.37, 0.09	0.10	0.87	0.77, 0.99	0.03
H3N2_A^2^	1.63	1.13, 2.37	0.01	1.11	1.04, 1.17	0.01
H3N2_B^c,e^	0.49	0.21, 1.10	0.08	0.83	0.68, 1.01	0.72
H3N2_B^2^	1.24	1.00, 1.53	0.04	1.04	0.99, 1.09	0.05
H1N1_C^c,f^	–	–	–	0.87	0.78, 0.96	0.01
H1N1_home^c,g^	1.19	0.53, 0.97	0.03	0.82	0.84, 0.98	0.01
*“*Low heterologous*”* H3N2 cluster	Baseline	–	–	Baseline	–	–
*“*High heterologous*”* H3N2 cluster^h^	2.66	0.86, 8.24	0.08	1.15	0.91, 1.44	0.22
Source			<0.01^**\\\**^			<0.01^**\\\**^
Source 1	5.5	1.54, 19.6	0.01	2.58	2.14, 3.11	0.01
Source 2	0.68	0.15, 3.08	0.62	1.34	1.02, 1.76	0.03
Source 3	5.5	1.54, 19.6	0.01	2.16	1.77, 2.65	0.01
Source 4	Baseline	–	–	Baseline	–	–
Source 5	1	–	–	1.96	1.39, 2.76	0.01
Study 2^b^						
H3N2_B^c,e^	0.36	0.10, 1.27	0.11	0.67	0.46, 0.98	0.04
H3N2_B^2^	1.15	0.96, 1.37	0.12	1.06	1.00, 1.12	0.03

OR: Odds Ratio, PWP-CP: Prentice, Williams and Peterson conditional probability.

^**\\\**^ *p*-value obtained by testing categorical variable using a partial likelihood test.

^a,b^Two distinct longitudinal studies were performed in nursery pigs.

^c^Estimates based on variable representing an hemagglutinin inhibition titer for a specific strain divided by 10 and then log2 transformed.

^d^Maternally derived antibodies as measured by using H3N2 strain heterologous to the resident strain in Study 1 (93.8% similarity in the hemagglutinin).

^e^Maternally derived antibodies as measured by using H3N2 strain heterologous to the resident strain in Study 1 (97.9% similarity in the hemagglutinin).

^f^Maternally derived antibodies as measured by using H1N1 virus broadly classified as classical H1N1 virus.

^g^Maternally derived antibodies as measured by using H1N1 strain detected in the Study 2 (i.e. homologous strain).

^h^Groupings obtained from the cluster analysis based on hemagglutinin inhibition titers of strains heterologous to the resident virus in Study 1.

When using the cluster membership based on heterologous H3N2 viruses, the results showed that the likelihood of having recurrent infection tended to be higher for pigs in cluster with high titers of heterologous H3N2 MDA present after weaning (OR = 2.66; *p* = 0.08; Table 5). It was also observed that the presence of MDA for PRRSV and *M. hyopneumoniae* was not associated with recurrent infection (*p* > 0.05). Using a partial likelihood ratio (LR) test, the sow source was declared as a significant risk factor in Study 1, but not in Study 2.

#### Stratified Cox’s regression for recurrent events

Results for the Cox’s regression model are presented in Table 5 for both studies. The results of the Cox’s regression models suggested that the hazard of IAV positivity was associated with the MDA against H3N2_A and H3N2_B viruses in a non-linear fashion which generally suggested that large increases in MDA leads to higher hazards of IAV infection regardless of the event number (Table 5). Existence of interactions between MDA titers for H3N2_A and strata was detected in one of the candidate models suggesting that higher hazard is expected for 3^rd^ and particularly 2^nd^ infection. However, we considered the latter model to be insufficiently robust due to the large numbers of variables considered (data not shown). Other variables associated with the presence of IAV are shown in Table 5. In Study 2, only H3N2_B MDA was statistically associated with IAV positivity. Presence of MDA for PRRSV and *M. hyopneumoniae* could not be detected as factors associated with presence of IAV for both studies (*p* > 0.05).

Using a partial likelihood ratio (LR) test, the sow source was considered as a significant risk factor in Study 1, but not in Study 2 (Tables 4 and 5), with pigs from sources 1, 3, and 5 having higher likelihood of infection in comparison to source 4.

## Discussion

Research on the factors that might drive influenza virus infection and maintenance in swine populations continues to be limited. Results of the present study demonstrate an endemic cyclical influenza virus infection in one nursery farm, which was a part of multi-site production systems that continuously experienced similar issues in multiple nursery herds.

None of the infection patterns observed in this study resembled the epidemic curve that would be expected when IAV is introduced into a susceptible closed population. Such epidemic curves have been frequently reported in the literature [7] and are characterized by abrupt and fast development of clinical signs and the underlying infection. The pattern observed in this study was more in line with clinical experiences in nursery populations described elsewhere [6]. Further studies are needed to document and elucidate with what frequency such continuous circulation of IAV occurs in swine. The presence of multiple swine influenza virus infections in a relatively short period of time and the number of recurrent infections observed in individual pigs open several questions about the epidemiology of IAV in pig herds. Answers to these questions could have direct implications for the design of infection control measures in pig herds and entire production systems.

The results of this study confirmed that individual animals could be detected with IAV on multiple occasions within a relatively short period of time. Recurrent infections with IAV in this age group have been previously reported under field conditions, in two different studies [23, 37]. In the former study, authors reported that the same viruses (H1N1 and H1N2) were detected in the same batch in two distinct outbreaks and even in the same pig. In the latter study, results indicated that more than 50% of the 62 animals followed were positive for IAV at least once and nine were infected on two different occasions with very similar viruses based on HA sequencing. In the present study the proportion of study pigs with recurrent positivity varied between 43.2% in Study 1, when H3N2 circulation was detected and 10.7% in Study 2 when H1N1 circulation was detected. From the limited results available so far in this study, it appears that individual animals were at least in some cases repeatedly detected with identical strain of H3N2 or H1N1 virus. More sequencing results need to be available in order to determine with certainty whether this was the exclusive manner in which pigs were recurrently positive in this study, or whether infections with different strains or subtypes could be detected as well. Regardless of the answer to the latter question, it is well established that pigs can shed IAV for up to 5–7 days after recovery [7, 38, 39]. Given the intensity of sampling, it is reasonable to assume that those pigs were likely showing a new infection with IAV every time that a positive result on virological testing was confirmed after a period with negative virological testing. Such a high proportion of pigs with recurrent infection was not expected, and it likely contributed to the endemic pattern of IAV circulation in this barn that occasionally reached a prevalence of 100%. This cyclical shedding pattern mimicked clinical description of respiratory signs before the study started. The exact reasons for positivity over time could be multiple, and we hypothesized that the following factors might contribute to such infection pattern: (1) influence of maternally-derived antibodies, or (2) environmental conditions, and (3) influence of other pathogens.

Presence of MDA and its association with recurrent infections and shedding of IAV have been previously reported [23, 40, 41]. Results from two experimental studies showed that pigs with MDA, coming from inoculated or vaccinated sows with a homologous strain, had a reduction in the expression of clinical signs after inoculation with the same strain [40, 41], but in the presence of strain-heterologous MDA or no MDA shedding period was longer [40]. Also active immunity was delayed or absent in the presence of MDA [41]. Results from a field trial, where sows were vaccinated with commercial vaccines containing three different IAV subtypes (H1N1, H1N2, H3N2), showed that increased shedding of H1N1 and H1N2 lasted longer in piglets with MDA. In addition, recurrent infections occurred during the nursery phase where pigs still had MDA circulating and those infected were found to carry the same IAV subtype presented in the vaccine (subtype-homologous) [23]. Also, results indicated that pigs in the follow up batches that were blood sampled in the finisher barn seroconverted showing that the absence of the MDA allowed the active immunity to build up. Thus, it was shown that the presence of MDA can interfere with humoral response and pigs were not fully protected against new infections [23] either with the same subtype or a different one, unless they presented MDA for an homologous subtype.

Results of the present study are consistent with previous literature in suggesting that high titers for a heterologous strain could influence IAV shedding and contribute to a higher risk of having recurrent infection. However, these conclusions are also subject to some uncertainties. First, the actual status of strain-homologous versus strain-heterologous within-subtype MDA was not easy to determine, due to the positive correlation between titers for different strains. Under field conditions encountered in this study, it was unknown whether high titers for a specific strain used in HI assay were indicative of exposure to this strain, or perhaps a consequence of cross-reactivity to a related similar strain. Because of that, cluster analysis was conducted on HI test results to facilitate natural groupings of pigs with similar serological results to multiple viruses within H3N2 and H1N1 subtypes. Results of ordinary logistic regression suggested that a cluster that contained pigs with high titers for multiple heterologous H3N2 viruses could indeed be a risk factor for recurrent infection. The second uncertainty is that the nature of exposure for different strains could also not be easily identified. There were five sow herds, but the adherence and stringency of vaccination protocol could not be assessed, and existence of endemic circulation of IAV in one or more of these sow herds was unknown. Both situations could result in sows and piglets with different immunity status with respect to the circulating strains in the nursery. In the second study, the level of MDA at entry to the nursery was high and more uniform for contemporary Ontario strains of H3N2 viruses than in the first study; however, the circulating strain at that time in the nursery was H1N1.

The same type of association between level of MDA and virological positivity could not be detected for H1N1 in this study. This could be because HA of H3N2 viruses in Ontario has been present since 2005 [18] and there has been more opportunity for H3N2 viruses to have point mutations and create larger antigenic diversity than H1N1 viruses. The H1N1 strain detected in this study was of the 2009 pandemic lineage, and these viruses show lower antigenic diversity at this point in time [17]. This situation might be not the same in other regions, or even production systems, if different strains of H1N1 are showing higher antigenic diversity. Final uncertainty is related to the curvilinear nature of the association between MDA and recurrent infection which was easier to interpret in the ordinary logistic regression. Despite some differences in the results for the two viruses, the latter model, when displayed on a probability scale, suggested that pigs with MDA titers above the cutpoint (i.e. > 40) had higher probability of recurrent infections than pigs that were at or below that cutpoint (i.e. 10–40). Nonetheless, pigs detected with titers below the detection limit (i.e. < 10) are expected to have slightly higher likelihood of recurrent infection compared to the pigs with titers ranging from 10 to 40. There was no obvious explanation for this finding except for the fact that animals influencing this association were located in the same pen, and therefore this finding could be a consequence of environmental conditions or other contributing factors in a single group of pigs.

With respect to environmental factors, results from the present study showed that ICC had high values for within-pen clustering. The latter finding, together with simple data visualization suggested that pigs within the same pen were more likely to shed the virus at the same time. This is in agreement with data from previous studies which showed that transmission between pens was lower when compared to transmission within pen [37] and that the number of pigs per water space by farm, i.e. quantity of waterers in the barn, was associated with higher likelihood of having positive results for IAV [42]. It was previously shown that transmission of the IAV is dependent on relative humidity and temperature [35, 43]. Both of these conditions could be associated with the viability of the virus, and relative humidity has been shown to influence the predominant types of droplets in the environment [44]. It has been shown that the size of droplets is important since large droplets that occur in areas with high relative humidity (≈100%) do not shrink easily and are more likely to fall to the ground [44]. Results from the present study are in agreement with those of previous findings since RH was high (daily mean between 66% and 76%), which could have favored large droplets and short-range transmission within pen. This, however, needs to be confirmed in field studies with concurrent measurements of climate, aerosol content and transmission risks within and between different natural groupings in swine barns.

Potential impact of other pathogens for IAV positivity was also attempted to be evaluated in this study. The presence of MDA for PRRSV and *M. hyopneumoniae* was not associated, in both studies, with positive virological results, a finding that was similar to other reports in the literature [23]. Despite the absence of an association between the presence of MDA for these two pathogens and IAV positivity, specific sow sources were associated with different aspects of IAV shedding. The variable representing sow sources was initially considered as an important confounding variable, which would have an impact on the MDA status of pigs. Nonetheless, the infection status of sow herds was also different with respect to several important swine pathogens, and this could have influenced the results of this study. In Study 1, sow source was identified as a risk factor with pigs from three sow-herds being under increased risk of having various aspects of IAV infection compared to sow-herd number 4, regardless of the analytical approach. This finding would suggest that factors common to a sow-herd (e.g., level of MDA for IAV or presence of other pathogens, host genetics, presence of other pathogens in weaned piglets, etc.) could contribute to the intensity of infection with IAV. However, in Study 2, sow source could not be identified as a significant risk factor, which perhaps suggests that whatever sow-source factors contributed to the IAV infection, they were likely of temporary nature. This difference in significance of sow sources between Study 1 and Study 2 may suggest that infection with other pathogens, or other “permanent” factors, in the sow herd did not play a large role in the intensity of infection with IAV during the nursery phase. Further studies are needed on the association between IAV shedding and the presence of MDA or the actual infectious agents for any of those diseases.

Interestingly, from all 400 and 300 pigs sampled at the very beginning of the two respective studies none were shedding the virus, although the sow-herds were almost certainly the source of the eventual IAV infection. Lack of detection could be due to low sensitivity of the cell culture in detection of pigs that shed low quantities of the virus. Results from other studies also showed that pre-weaned pigs are important in maintaining disease circulation, although the virus could be present at levels below the detection limits [45]. A possibility exists that pigs indeed were virus negative at arrival time, and that the virus was introduced by infected humans, or asymptomatic carriers [37]; or from environment, or from any remaining pigs from a previous batch that could be present in a separate barn. The likelihood for these possibilities are low because similar outbreaks were occurring repeatedly, the barn was disinfected and washed between batches, and only a small number of pigs could have been present on the site, but never in the same barn, and only under unusual circumstances.

Some limitations of the study included inability to build multivariable models because of low number of pigs and non-linear relationships that were addressed through quadratic effects, and relatively long time between measurements of individual pigs. Survival analysis that was used in this study could not be easily adjusted for such interval censoring, but we opted to use it because it was important to explore factors contributing to recurrent infections from multiple perspectives. Following animals from the farrowing would have been beneficial, but was not possible because of logistical reasons and biosecurity protocols. Another limitation, concerning isolation of viruses, could be associated with low sensitivity of the test performed, if this was the scenario. The selection of this test was based on the fact that we wanted to have confirmation that the viruses were viable and capable of causing disease. Another option would be to perform a test to detect nucleic acid, but many of the inferences that we made would not be possible. It could also be argued that different viruses might be present and the dominant one would impede the culture of another one. Such events should be explored in future studies by appropriate application of deep sequencing methods directly from diagnostic specimens. Despite such limitations, this study provides useful insight into the epidemiology of IAV in the contemporary swine production systems.

In conclusion, results of this study suggested that nursery pigs could be infected on multiple occasions with IAV. Between 10 and 43% of pigs had recurrent infection with H1N1 and H3N2 viruses in the two different studies, respectively. This contributed to a cyclical pattern of IAV positivity in the nursery barn. Virus shedding, including recurrent infections, was associated with high level of HI titers for strains that were heterologous to the resident virus, but only for the H3N2 strain. However, the presence of high heterologous immunity is not likely to explain all recurrent infections because pigs with low heterologous infections were also noticed to be recurrently infected. A high degree of within-pen clustering was observed, suggesting that transmission within a pen played an important role. A microclimate that favored large droplets and short-distance transmission possibly contributed to this within pen transmission. Prolonged or recurrent IAV infections could be of great importance when trying to control IAV infection in nursery barns.

## Additional file

**Additional file 1.** **Spearman correlation coefficients with statistical significance adjusted for multiple comparisons using Šidák approach.**

## Abbreviations

IAV: influenza A virus
AIAO: all-in all-out
PBS: phosphate-buffered saline
MDCK: Madin-Darby canine kidney
CPE: cytopathic effect
HI: hemagglutination inhibition assay
ELISA: enzyme-linked immunosorbent assay
PRRSV: porcine reproductive and respiratory syndrome virus
MDA: maternally-derived antibodies
ICC: intra-cluster correlation coefficient
PWP-CP: Prentice, Williams and Peterson conditional probability
RH: relative humidity
AH: absolute humidity
CSV: comma separated value
SD: standard deviation
