# Supplementary material for: Longitudinal study of influenza A virus circulation in a nursery swine barn
Source: Vet Res. 2017 Oct 10;48:63. doi: 10.1186/s13567-017-0466-x (PMC5634873; doi:10.1186/s13567-017-0466-x)
Supplement: Supplementary file 1 — Additional file 1. Spearman correlation coefficients with statistical significance adjusted for multiple comparisons using Šidák approach. [file 13567_2017_466_MOESM1_ESM.docx]

**Additional file 1.** Spearman correlation coefficients with statistical significance adjusted for multiple comparisons using Šidák approach.

| **Study 1** | | | | | | | |
| --- | --- | --- | --- | --- | --- | --- | --- |
|  | | H3N2_C | H3N2_A | H3N2_B | H3N2_H | H1N1_P | H1N1_C |
| H3N2_C | | 1.00 |  |  |  |  |  |
| H3N2_A | | 0.76 | 1.00 |  |  |  |  |
| H3N2_B | | 0.61 | 0.64 | 1.00 |  |  |  |
| H3N2_H | | 0.43 | 0.45 | 0.51 | 1.00 |  |  |
| H1N1_P | | 0.40 | 0.42 | 0.38 | 0.18* | 1.00 |  |
| H1N1_C | | 0.50 | 0.49 | 0.25^*^ | 0.14* | 0.41 | 1.00 |
| H1N1_H | | 0.50 | 0.44 | 0.46 | 0.51 | 0.45 | 0.51 |
| H1N2 | | 0.82 | 0.80 | 0.64 | 0.39 | 0.42 | 0.49 |
| ^*^ | Correlation not statistically significant (P>0.05) | | | | | | |

| **Study 2** | | | | | | | |
| --- | --- | --- | --- | --- | --- | --- | --- |
|  | | H3N2_C | H3N2_A | H3N2_B | H3N2_H | H1N1_P | H1N1_C |
| H3N2_C | | 1.00 |  |  |  |  |  |
| H3N2_A | | 0.78 | 1.00 |  |  |  |  |
| H3N2_B | | 0.63 | 0.60 | 1.00 |  |  |  |
| H3N2_H | | 0.67 | 0.53 | 0.69 | 1.00 |  |  |
| H1N1_P | | 0.42 | 0.33* | 0.13* | 0.13* | 1.00 |  |
| H1N1_C | | 0.38 | 0.34* | 0.12* | 0.11* | 0.69 | 1.00 |
| H1N1_H | | 0.14* | 0.24* | −0.11* | −0.08* | 0.68 | 0.61 |
| H1N2 | | 0.84 | 0.65 | 0.66 | 0.66 | 0.43 | 0.39 |
| ^*^ | Correlation not statistically significant (*p* > 0.05) | | | | | | |
